# Promising performance of chemically exfoliated Zr-doped MoS_2_ nanosheets for catalytic and antibacterial applications

**DOI:** 10.1039/d0ra02458a

**Published:** 2020-05-29

**Authors:** M. Ikram, R. Tabassum, U. Qumar, S. Ali, A. Ul-Hamid, A. Haider, A. Raza, M. Imran, S. Ali

**Affiliations:** Solar Cell Applications Research Lab, Department of Physics, Government College University Lahore 54000 Punjab Pakistan dr.muhammadikram@gcu.edu.pk; Department of Physics, Riphah Institute of Computing and Applied Sciences (RICAS), Riphah International University 14 Ali Road Lahore Pakistan; Department of Gynaecology & Obstetrics (Unit–III), Jinnah Hospital Lahore Punjab 54000 Pakistan; Center for Engineering Research, Research Institute, King Fahd University of Petroleum & Minerals Dhahran 31261 Saudi Arabia; Department of Clinical Medicine and Surgery, University of Veterinary and Animal Sciences Lahore 54000 Punjab Pakistan; State Key Laboratory of Chemical Resource Engineering, Beijing Advanced Innovation Centre for Soft Matter Science and Engineering, Beijing Engineering Center for Hierarchical Catalysts, Beijing University of Chemical Technology Beijing 100029 China

## Abstract

Nanostructured materials incorporated with biological reducing agents have shown significant potential for use in bactericidal applications. Such materials have also demonstrated considerable efficacy to counter effects of chemical toxicity. In this study, nanostructured molybdenum disulfide (MoS_2_) was doped with various concentrations (2.5, 5, 7.5, 10 wt%) of zirconium (Zr) using a hydrothermal route in order to assess its antimicrobial and catalytic potential. Doped and control samples were characterized with various techniques. X-ray diffraction (XRD) analysis confirmed the presence of the hexagonal phase of MoS_2_ and identification of various functional groups and characteristic peaks (Mo bonding) was carried out using FTIR spectra. Micrographs obtained from FESEM and HR-TEM showed a sheet-like surface morphology, while agglomeration of nanosheets was observed upon doping with nanoparticles. To seek further clarity regarding the layered features of S–Mo–S planes, the defect densities and electronic band structure of pure MoS_2_ and doped MoS_2_ samples were investigated through Raman analysis. Optical properties of Zr-doped MoS_2_ nanosheets were assessed using a UV-vis spectrophotometer and the results indicated a red-shift, *i.e.*, movement of peaks towards longer wavelengths, of the material. Dynamics of migration and recombination of excited electron–hole pairs were investigated using PL spectroscopy, which was also used to confirm the presence of exfoliated nanosheets. In addition, the synthetic dye degradation potential of pure and doped samples was investigated in the presence of a reducing agent (NaBH_4_). It was noted that doped MoS_2_ showed superior catalytic activity compared to undoped MoS_2_. The nanocatalyst synthesized in this study exhibited enhanced antibacterial activity against *E. coli* and *S. aureus* at high concentrations (0.5, 1.0 mg/50 μl). The present study suggests a cost-effective and environmentally friendly material that can be used to remove toxins such as synthetic dyes and tannery pollutants from industrial wastewater.

## Abstract 2

Nanostructured materials incorporated with biological reducing agents have shown significant potential for use in bactericidal applications.

## Graphical abstract

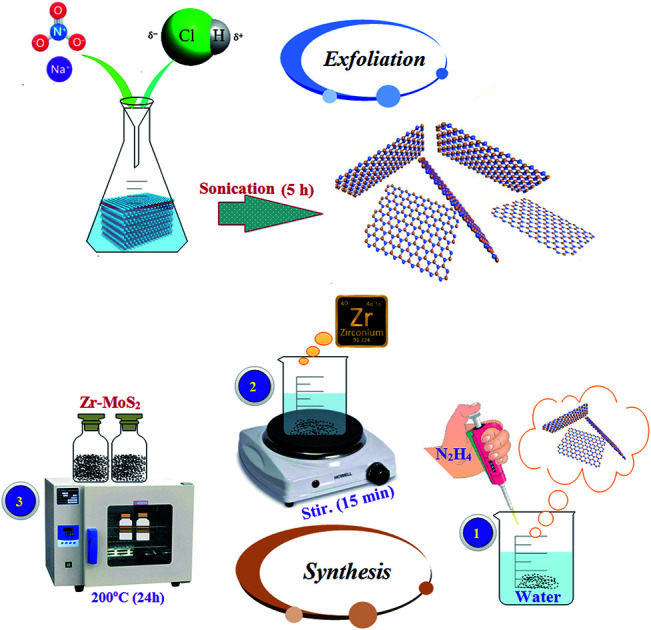

## 1. Introduction

Lack of availability of clean potable water is an environmental concern that is shared at a global level. As the world's population continues to grow, an estimated 750 million people lack access to safe and clean drinking water. Around 97.5% of the earth's water reservoir is salty which leaves only 2.5% water that is held in reserve for the sustenance of humankind.^1,2^ Untreated wastewater emanating from various industries such as, cosmetics, paper, textile and chemical manufacturing not only serves to pollute and diminish an already limited supply of clean water but also creates a hazard for the environment and living species alike.^3^ In view of above, it has become of utmost importance to devise innovative ways to purify polluted water in order to make its availability more widespread especially in developing countries where this issue requires immediate attention. Various techniques have been used for this purpose, for instance, photocatalysis, precipitation, reverse osmosis (RO), and adsorption. Among these techniques, adsorption has received widespread attention owing to its simple operation, low energy consumption, high efficiency and cost-effectiveness. It has been shown especially effective for its use in removing dye pollutants from industrial effluence.^4,5^ In this context, adsorption properties of carbon, silica gel (SiO_2_), polymers and activated alumina adsorbents are frequently studied. Carbon is regarded as an excellent adsorbent for the removal of several pollutants in aqueous solutions.^6^ Furthermore, some new carbon materials have been developed including carbon nanotubes (CNTs)^7^ and carbon nanofibers (CNFs)^8^ which show a high degree of adsorption during purification process of water. However, the efficacy of these materials is limited to only certain types of pollutants *e.g.*, organic compounds and metal ions. Therefore, there is a continuous need to produce materials that possess improved capability of adsorption for use in applications developed for the treatment of polluted water.

A possible solution may exist in the form of new generation materials produced at nanoscale. Recently, two dimensional (2D) nanomaterials have attracted considerable attention in the area of catalysis,^9^ photocatalysis, optoelectronics, and energy-related fields (ERF)^10^ due to its high chemical stability and large surface area.^11^ Among these, MoS_2_ is an efficient transition metal dichalcogenide (TMD) that has a layered structure similar to graphene. MoS_2_ nanomaterial is composed of three stacked atomic layers *i.e.* Mo atomic layer sandwiched between two sulfur (S) layers held together through weak van der Waals forces across the layer.^12^ MoS_2_ layers are characterized by short interlayer distance and strong chemical bond forms with each layer which gives rise to high catalytic activity. In this regard, it is envisaged that due to its potentially strong catalytic and photocatalytic activity, exfoliated MoS_2_ nanosheets could prove effective for use in applications developed for environmental remediation^13,14^.

Another endemic problem the modern world is beginning to face is the development of resistance to antibiotic drugs in bacteria. The Infectious Diseases Society of America (IDSA) has stated that the worldwide infection of most dreadful bacterial species called ESKAPE pathogens has developed strong antibiotic resistance^15^. A number of deaths have been recorded worldwide caused by these pathogens. Every year, *Escherichia coli* (*E. coli*) a family member of ESKAPE pathogen causes death of 1.3 million children under the age of five due to diarrhea, which is mainly transmitted from contaminated water. To mitigate this enormous loss, a continuous effort is required to develop new antibiotics that could control the bacterial growth of ESKAPE pathogens. In this respect, 2D materials are potential candidates due to their superior antibacterial activity, *e.g*., graphene oxide (GO) and chemically exfoliated MoS_2_ have been shown to work efficiently against several microorganisms^16^. As MoS_2_ has a large surface area, it generates strong surface charge mobility and conductivity which might facilitate bacteria adhesion^17^. These materials may be duly effective for use in biomedical applications that do not release any toxic biocide into the environment^18^. MoS_2_ has generated great interest due to its antimicrobial activity against pathogens Gram-positive (*S. aureus*) and Gram-negative (*E. coli*)^19^. MoS_2_ nanocomposites have exhibited rapid and effective reaction for killing bacteria *in vitro* and wound disinfection *in vivo*^20^.

Various nanocatalysts such as, Bi, Co, and Cu have been employed so far in an effort to enhance the efficiency of catalytic activity. These catalysts are capable of degrading MB in 3–4 minutes, which is a notable accomplishment^21,22^. In this study, Zr nanoparticles were used as a catalyst to enhance the catalytic and antimicrobial efficiency of MoS_2_ prepared through hydrothermal technique. The study revealed that Zr-doped MoS_2_ nanosheets were significantly more effective in removing organic dye (methylene blue) compared to other transitions metals. For this reason, the synthesize product can be considered as a superior candidate for use in antimicrobial applications developed in the near future.

## 2. Experimental details

### 2.1 Chemicals

Molybdenum disulfide (MoS_2_, 99.8%) and sodium borohydride (NaBH_4_) were procured from Sigma-Aldrich (Germany) and hydrogen chloride (HCl, diluted 37%) was acquired from Analar chemical lab. Sodium nitrate (NaNO_3_) and zirconium nitrate hydrate (Zr(NO_3_)_4_·H_2_O) were received from BDH Laboratories (United Kingdom). Bacterial growth media were of analytical grade provided by TM Media, (Titan Biotech Ltd, India). All chemicals were used in this work without additional purification.

### 2.2 MoS_2_ exfoliation process

**Fig. 1:**
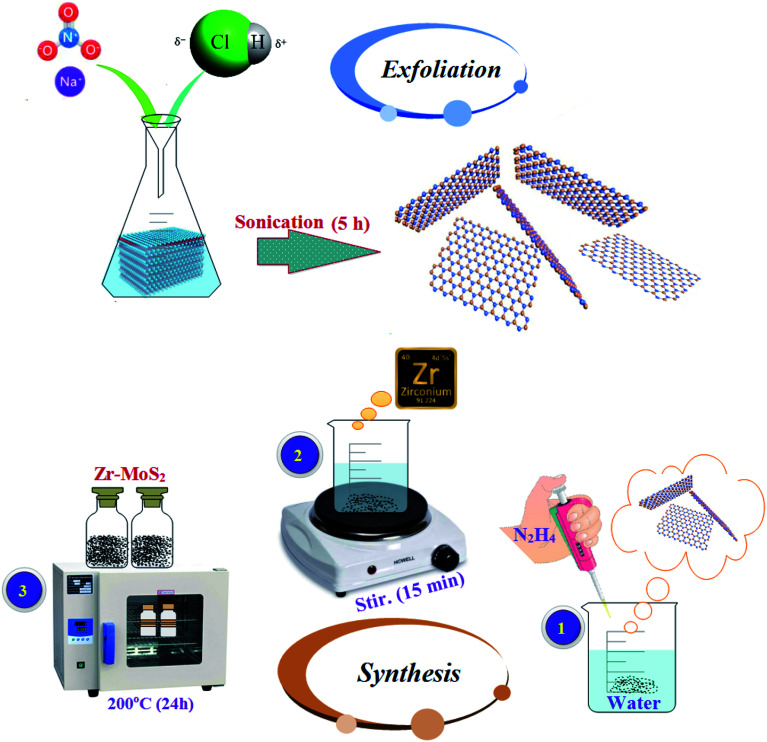
Various steps of chemical exfoliation and synthesis of Zr-MoS_2_.

MoS_2_ nanosheets were synthesized *via* chemical exfoliation route as depicted in Fig. 1. Sodium nitrate (6 g) was dissolved in 16 ml of 37% diluted HCl in single-neck round flask. Later, bulk MoS_2_ (1.2 g) was added to the above-mentioned solution, and the reaction was quenched with water. Prepared solution was sonicated at 30 °C for 5 hours with toxic gas collection setup. Obtained supernatant fraction was centrifuged at 6000 rpm for 30 minutes. Finally, grey black precipitated MoS_2_ nanosheets were collected.^2^

### 2.3 Synthesis of Zr-doped MoS_2_ nanosheets

Hydrothermal synthesis was used to prepare the desired concentrations of Zr-doped MoS_2_ nanosheets as shown in Fig. 1. In this method, various concentrations (25, 50, 75, 100 mg) of Zr nitrate hydrate (Zr(NO_3_)_4_·H_2_O) with 5 ml hydrazine hydrate (N_2_H_4_) as a reducing agent were added in 80 ml DI water under constant stirring at 70 °C for 15 minutes and then transferred to 100 ml of tightly sealed Teflon-lined stainless steel autoclave. In this process, Zr nanoparticles develop bonds with the host material due to high pressure and temperature, thus resulting in the formation of a new product Zr-MoS_2._ In order to complete the reaction, the solution was kept in the autoclave at 200 °C for 24 hours. Finally, prepared black solution was dried at 200–250 °C for complete elimination of impurities such as NH_3_/NH_4_^+^ ions. These impurity ions are released as byproducts of hydrazine.^23^

### 2.4 Materials characterization

Structural and phase information of doped sample was obtained with the help of X-ray diffraction (XRD). PANalytical X'pert equipped with Cu-Kα radiation (*λ* ∼ 0.154nm) was used by varying diffracted angle 2*θ* from 10° to 65° for this purpose. The presence of various functional groups was identified with FTIR PerklinElmer spectrometer, and optical properties were examined using UV-vis Spectrophotometer (Genesys 10S) set in the range of 250–350 nm. Surface morphology and interlayer spacing was investigated using FESEM, JSM-6460LV and HRTEM Philips CM-30 and JEOL JEM 2100F. To confirm the presence of MoS_2_ flakes, Raman spectra were recorded on Renishaw with Reflex confocal Raman microscope operated at a wavelength of 532 nm (6 mW) laser. Photoluminescence spectra of as-prepared and doped samples were recorded using spectrofluorometer (JASCO, FP-8300).

### 2.5 Catalytic potential

**Fig. 2:**
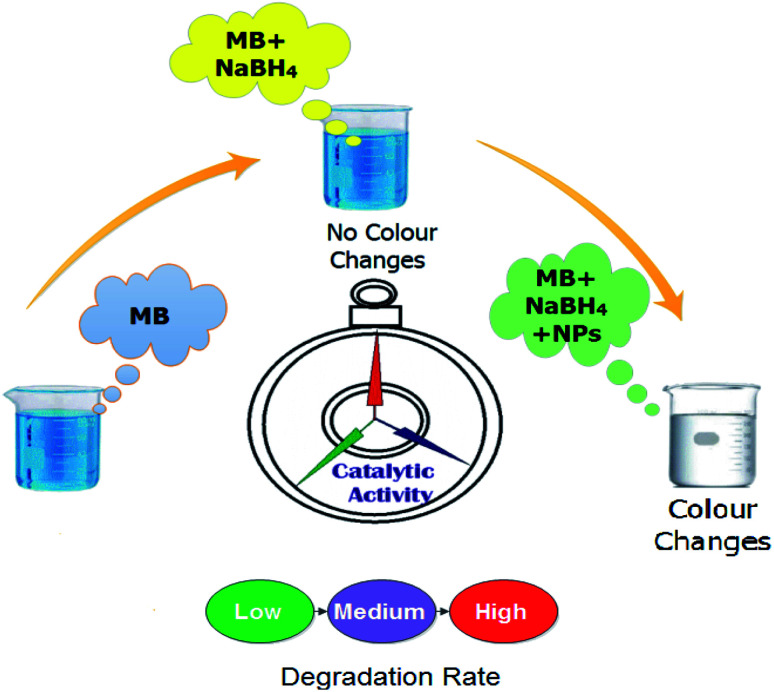
Schematic diagram of the experimental procedure adopted to measure catalytic activity.

The catalytic activity of the undoped and doped MoS_2_ nanosheets was assessed in terms of degradation of methylene blue (MB) which acts as an oxidizing agent in the presence of a reducing agent NaBH_4_. In this study, the synthesized product acts as a catalyst. The amount of catalyst used in the reaction is a crucial factor during the regulation of catalytic activity. With increasing concentration of the catalyst, the degree of dye degradation is enhanced since a catalyst minimizes the activation energy required for the reaction to take place. With regards to the experiment method used in the present study, freshly prepared 400 μl solution of sodium borohydride (NaBH_4_) (0.1 M) was added in 3 ml aqueous methylene blue (10 ppm) in a quartz cell. This was followed by the addition of 400 μl of Zr-doped nanosheets with specific concentrations (0 : 1, 0.025 : 1, 0.050 : 1, 0.075 : 1, 0.10 : 1) to the solution. Decolorization and variations in absorption intensity of the dye represented the reaction rate over periodic intervals as shown in Fig. 2. Reaction without nanocatalyst is referred to as the one with reference sample. UV-vis spectrophotometer was employed to acquire absorption spectra at various intervals in the range of 200–750 nm (Fig. 8).

### 2.6 Isolation and identification of *S. aureus* and *E. coli*

Caprine mastitic milk samples collected from various farms of Punjab, Pakistan were cultured on 5% sheep blood agar. The cultured samples were incubated for 24 hours at 37 °C and the characteristic colonies thus obtained were further streaked on manitol salt agar (MSA) and MacConkey agar (MA) in order to isolate purified *Staphylococcus aureus* (*S. aureus*) and *Escherichia coli* (*E. coli*), respectively in triplets. Identification of purified colonies proceeded through Gram's staining, morphological study, and biochemical tests (*i.e.*, catalase and coagulase tests).

### 2.7 Antibacterial activity

Antibacterial evaluation of Zr-doped MoS_2_ nanosheets was conducted using Gram-positive (G +ve) *S. aureus* and Gram-negative (G −ve) bacteria *E. coli* directly isolated from caprine mastitis. *In vitro* antibacterial efficacy was assessed through agar well diffusion test by swabbing *S. aureus* and *E. coli* isolates on MSA and MA, respectively. Bacterial suspensions of 1.5 × 10^8^ CFU ml^−1^ (0.5 Mc-Farland standards) were swabbed on Petri dishes and wells containing 6 mm diameter were formed using a sterile cork borer. Various concentrations of Zr-doped MoS_2_ (0.5 mg/50 μl) and (1.0 mg/50 μl) were loaded into each well and compared with ciprofloxacin (0.005 mg/50 μl) and DIW (50 μl) as positive and negative control, respectively under aseptic conditions. The loaded Petri dishes were incubated at 37 °C for 24 hours and antibacterial evaluation of Zr-doped MoS_2_ was conducted with Vernier caliper that measured inhibition zones in millimeter (mm).

#### 2.7.1. Statistical analysis

The antibacterial efficacy, calculated in terms of inhibition zone diameters (mm), was statistically analyzed by one-way analysis of variance (ANOVA) using SPSS 20.

## 3. Results and discussion

**Fig. 3:**
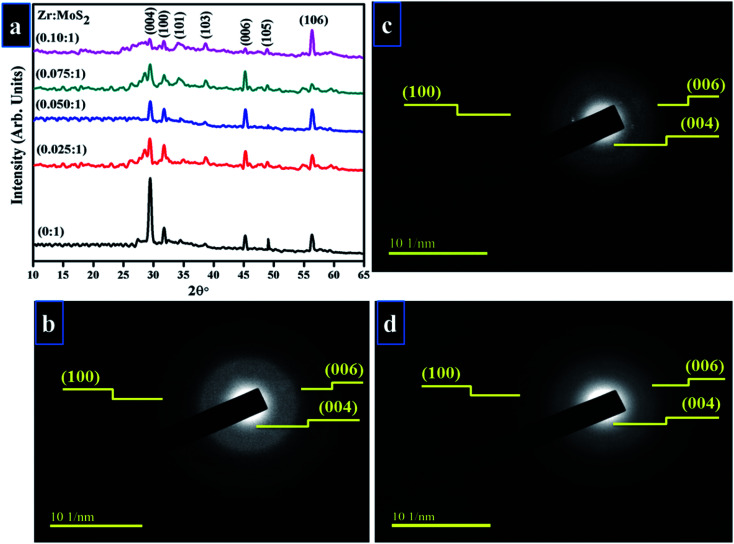
(a) XRD pattern (b–d) SAED rings of (b) 0 : 1 (c) 0.050 : 1 (d) 0.10 : 1 samples.

Crystallinity and phase purity of Zr-doped MoS_2_ nanosheets were examined using XRD technique (Fig. 3a). Detectable diffraction peaks observed at 2*θ* = 29.42°, 32.78°, 34.47°, 38.59°, 45.39°, 49.20°, 56.30° correspond to (004), (100), (101), (103), (006), (105) and (106) planes respectively.^2,24,25^ Observed reflections reveal hexagonal phase without impurity peak, which correlated well with JCPDS card no. 00-37-1492.^20^ Low-intensity diffraction peaks indicate low crystallinity of Zr-MoS_2_ while the presence of (004) face possibly resulted from partial restacking that occurred when the samples were dried during synthesis.^26,27^ Peaks observed at (100), (103) and (105) are distinctive MoS_2_ peaks, representing crystal planes that are well crystalized and belong to the hexagonal phase of MoS_2_.^28,29^ Interlayer spacing of nanosheets at 32.7° (100) was evaluated with Bragg's equation 2*d* sin *θ* = *nλ*, and was found to be 0.27 nm, which also corroborates the information obtained from HR-TEM images discussed later in Fig. 6.^30^ SAED analysis of un-doped and doped MoS_2_ was undertaken in a small region inside sphere while a large area exhibits the whole sphere as shown in Fig. 3b–d. Diffraction rings within SAED pattern were indexed as (004), (006) and (100) crystal planes belonging to MoS_2_ hexagonal phase, which agreed well with the XRD results.

**Fig. 4:**
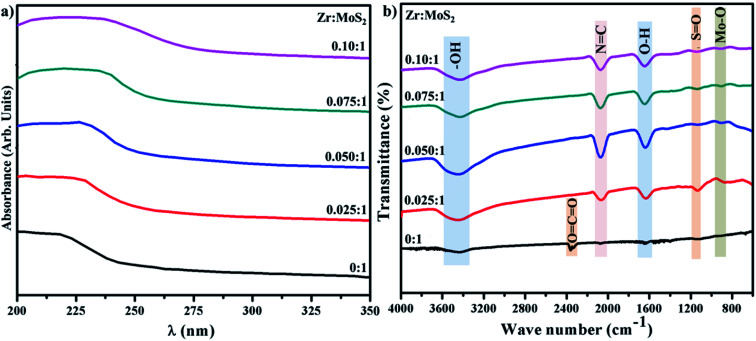
(a) UV-vis analysis (b) FTIR spectra of control sample and Zr-doped MoS_2_.

UV-vis spectroscopy was applied to investigate the optical properties of synthesized Zr-doped MoS_2_ nanosheets, as shown in Fig. 4a. MoS_2_ nanosheets exhibit broad light absorption in the visible range owing to its intrinsic narrow band gap coupled with multilayer nanostructure. The pristine sample shows a light UV response with an absorbance band at around 225 nm. In case of Zr-doped nanosheets, absorption range is increased with a slight redshift (toward longer wavelength) in UV zone. Redshift is caused by the presence of Zr nanoparticles, which indicates a possible charge–transfer transition between Zr and MoS_2_.^31^

FTIR spectra were acquired to identify various functional groups present in the control and doped sample in the range of 4000–400 cm^−1^ as illustrated in Fig. 4b. Broad and sharp peaks observed at 3454 and 1637 cm^−1^ belong to symmetrical –OH stretching-vibration and water bending modes.^32^ The transmittance band appearing around 2170 cm^−1^ in the doped sample is attributed to N=C.^33^ Peak observed in control sample at ∼2358 cm^−1^ represent carbon double bond (CO_2_), while band at 1124 cm^−1^ is assigned to S=O bonding vibrations.^33,34^ Moreover, peak occurring at ∼903 cm^−1^ is related to Mo–O vibration which is in agreement with the formation of MoS_2_ that possesses partially-oxidized edges.^35^

**Fig. 5:**
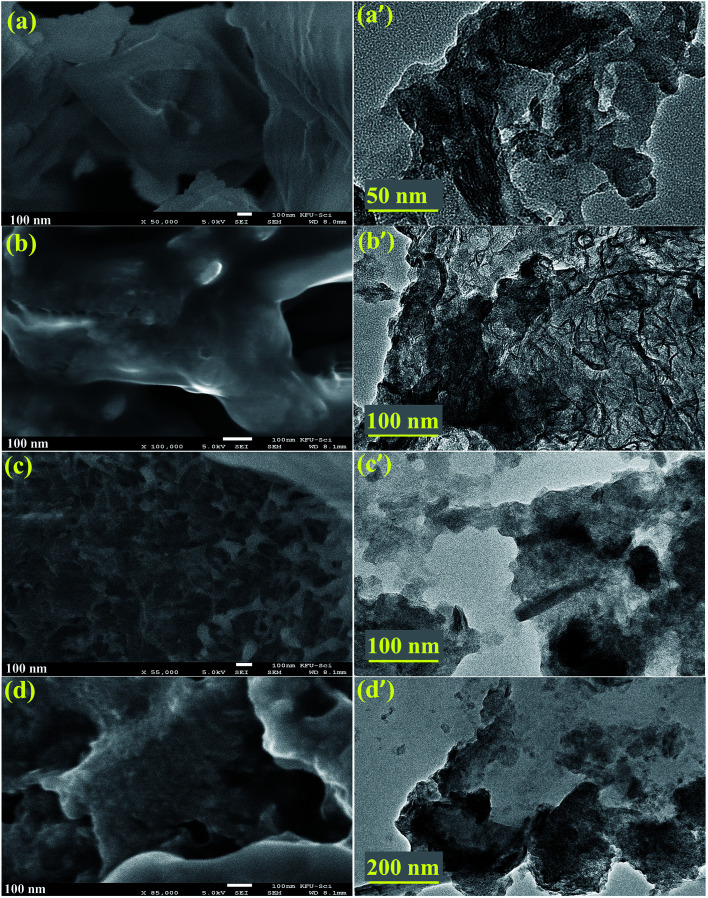
(a–d) FESEM micrographs of Zr-doped MoS_2_ (0 : 1, 0.05 : 1, 0.075 : 1 and 0.1 : 1 samples respectively) and (a′–d′) corresponding HR-TEM images.

The morphology and structure of pure and doped MoS_2_ was examined using FESEM and the results are shown in Fig. 5a–d. From the images, it can be seen that pure MoS_2_ showed nanosheets of plane-surface morphology with a thickness and lateral length of ∼100 nm (Fig. 5a). Upon doping with Zr-NPs, nanosheets depict the same morphology and lateral length, however the sheet-surface exhibits a rougher appearance (see Fig. 5c). It can also be observed that many nanoparticles are anchored on the surface of nanosheets (Fig. 5b–d). Morphology and microstructure of pure and doped MoS_2_ were further characterized with HR-TEM as illustrated in Fig. 5(a′–d′). HR-TEM results verify that MoS_2_ samples possess layered sheet-like morphology and are somewhat agglomerated. On the other hand, Zr-doped samples present a layered structure with delaminated sheets and dark spots provide evidence for the presence of Zr on MoS_2_ nanosheets.^36,37^

**Fig. 6:**
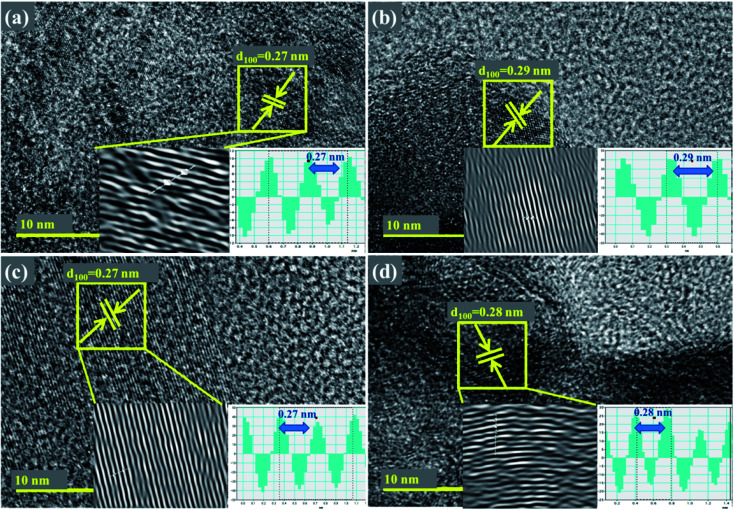
(a–d) HR-TEM images showing d-spacings of Zr-MoS_2_ (a) 0 : 1 (b) 0.050 : 1 (c) 0.075 : 1 (d) 0.10 : 1 samples.

Detailed characterization of nanosheets were further undertaken with HR-TEM up to 10 nm as depicted in Fig. 6, where surface morphology was observed to exhibit a layered structure in the synthesized products. Integral lattice of sheet-like MoS_2_ exhibited no defects or deformation in the samples prepared through exfoliation route.^38^ HR-TEM results display filtered micrograph and Fast Fourier Transform [FFT] of the selected area specified by yellow-square in Fig. 6a–d, which provides structural information at a higher resolution. For pure sample, interlayer spacing was calculated as 0.27 nm, which is consistent with the reported data and XRD results.^30,39^ Upon doping with Zr-NPs, *d*-spacing of 0.29, 0.27 and 0.28 nm were measured.

**Fig. 7:**
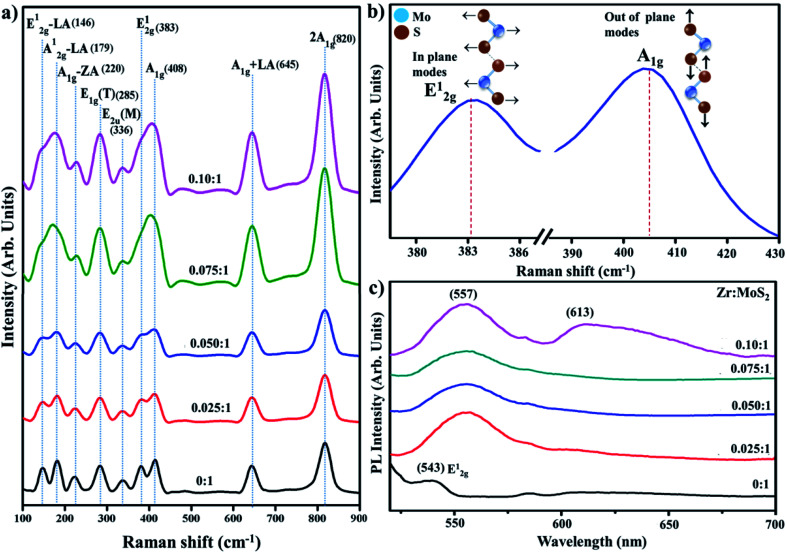
(a) Raman spectra of prepared samples (b) characteristic modes of MoS_2_ (c) PL spectra.

Raman spectroscopy was employed to probe structural properties with regards to the number of layers, defects densities, and electronic band structure. Raman scattering was performed in the range of 100–900 cm^−1^ (see Fig. 7a).^40^ In the observed spectra of the prepared products, peaks are located at 146, 179, 220, 285, 336, 383, 408, 645 and 820 cm^−1^, which is consistent with previously reported literature.^41–43^ Two characteristics peaks were exhibited by all samples, *e.g.*, first-order active modes that exist in most of the reported Raman studies of MoS_2_. These peaks were assigned as E_2g_ (383 cm^−1^) indicating in-plane vibrational mode and A_1g_ (408 cm^−1^) that signify out-of-plane vibrations, these modes are attributed to the layered structure of S–Mo–S planes.^41^ Due to the dependence of these two modes on the number of layers of MoS_2_, sharpness of peaks is slightly decrease with an increase in the inserted species may be attributed to laser-induced heating phenomenon.^40,43^ Peaks around 145 and 179 cm^−1^ are assigned to E_2g_ – LA(M) and A_1g_(M) – LA(M) phonon mode, respectively.^41,44^ The 645 cm^−1^ band is a combination of LA(M) frequency and A_1g_ mode, while the 176 cm^−1^ band arises due to subtraction of LA(M) from A_1g_ frequency (408 cm^−1^), as shown in Fig. 7b.^41^ Energy of LA(M) phonon determined from the procedure is somewhat larger than the value determined from the energy of 2LA(M) peak in non-resonant spectrum.^45^ The peak observed at 820 cm^−1^ is attributed to the second second-order spectral region (2A_1g_).^32^

Photoluminescence (PL) was employed to elucidate the electron–hole pair recombination process of the control and Zr-doped sample, as shown in Fig. 7c. It is an effective way to characterize 2D MoS_2_ as its PL effect is strongly associated with its electronic-band structure.^46^ Bulk MoS_2_ has 1.2 eV of indirect band gap whereas exfoliated MoS_2_, which makes a transition from indirect band gap to direct band gap of 1.8 eV, has enhanced PL owing to relaxation-of-excitons at Brillouin zone *K* point.^47^ It is observed in 2D MoS_2_ nanosheets that an increase in excitation wavelength proceeds to a redshift in PL spectrum with *λ*-range from 480 to 540 nm. This observation is associated to the excitation dependent PL is related with poly-dispersity of two-dimensional nanosheets.^48^ Observed peak around 543 nm is attributed to E_2g_ vibrational mode associated with in-plane vibrations of S atoms, as also discussed in Raman spectra.^49^ Upon doping with Zr, two absorptions band appeared at ∼557 and 613; peak at 557 nm can be attributed to nanosheets with lateral dimensions of a few tens of nm.^50^ The peaks located at 613 nm signify-B direct excitonic-peak.^51^ The above-stated results acquired from the analysis of MoS_2_ nanosheets suggest quantum confinement effect.

**Fig. 8:**
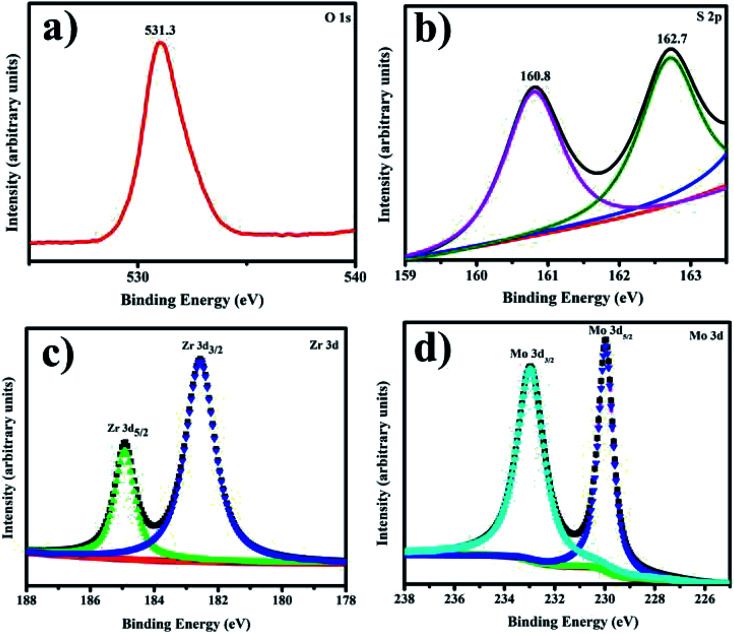
(a–d) XPS spectra of prepared samples (a) O 1s (b) S 2p (c) Zr 3d (d) Mo 3d.

XPS analysis shows the composition of Zr-doped MoS_2_ nanosheets with the presence of O, C, Zr, and Mo as illustrated in Fig. 8a–d. The Mo 3d spectra were recorded and deconvoluted as presented in Fig. 8a–d. All observed peaks of O, C, Zr and Mo accord with existing reports.^56–58^ The two peaks present in the spectra of O1s region confirmed the existence of two types of oxygen species on the surface, see Fig. 8a. The peak positioned at 531.3 eV in Fig. 8a could be assigned to silanol group for O1s species.^59^ The two peaks seen at 162.7 and 160.8 eV correspond to the presence of polysulfide in MoS_2_ as depicted in Fig. 8b. Zr 3d_3/2_ and Zr 3d_5/2_ were observed at 182.5 eV and 184.7 eV, respectively, see Fig. 8c. In catalyst, Zr binding energy is greater than pure Zr metal (180.0 eV). It is lower than 182.9 eV in ZrO_2_ and similar with ZrOx at 181.4 eV.^60^ Conclusively, it is clear that Zr cations were effectively incorporated within MoS_2_.^59^ In Fig. 8d, Mo 3d spectra verified various oxidation states of Mo atoms as Mo^4+^, Mo^5+^ and Mo^6+^.^61^ Mo 3d_5/2_ and Mo 3d_3/2_ binding energies indicate different oxidative states of Mo ions in the synthesized material.^62^

**Fig. 9:**
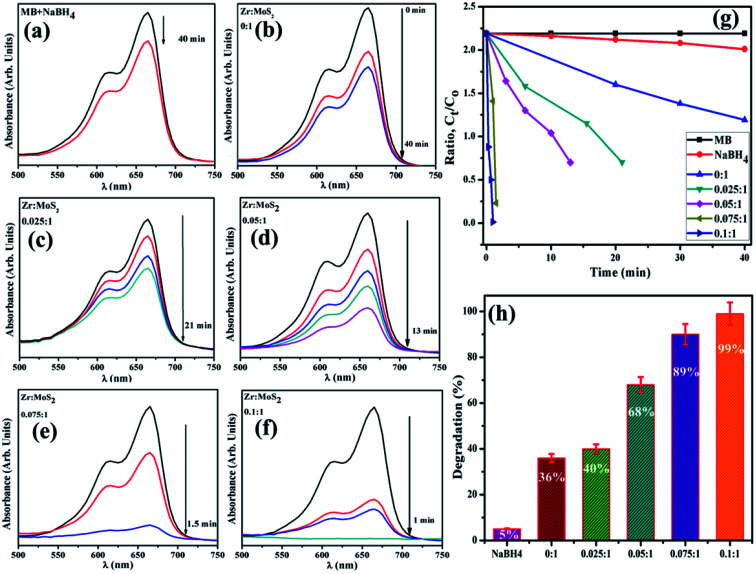
(a–f) Time dependent UV-vis spectra of dyes reduction (g) degradation-ratio with time (h) comparison of degradation (%) efficiency of different concentration of Zr-doped MoS_2_.

To assess the potential of Zr-doped MoS_2_ nanosheets, MB was used as contaminant to determine the catalytic activity in environmental remediation process. Catalytic degradation of organic dye (MB) was investigated using sodium borohydride (NaBH_4_) as a reducing agent while Zr-doped MoS_2_ was employed as a nanocatalyst. The reaction was examined in the range of wavelength 500–750 nm using UV-vis spectrometer. In an aqueous medium, the absorption peak of MB was observed at 665 nm. Reducing ability of NaBH_4_ with MB and MoS_2_ was not observed to be significant, as it degraded 5% and 36% of MB only in 40 min respectively, as presented in Fig. 9a. Concentration of methylene blue continuously decreased within an increase in doping concentration of Zr in MoS_2_ and the maximum catalytic efficacy was noted for 0.1 : 1 (see Fig. 9f). In the presence of a catalyst, reduction of the dye was accelerated, which is represented by a sharp decrease in the absorption peak of MB. Catalysts facilitate electron relay from donor (BH_4_) to acceptor (MB), in which case catalysts accept electrons from BH_4_ ions and transfer them to MB.

It was observed that the sample doped with a lower concentration of dopant (0.025 : 1 sample) showed incomplete reduction of dye with 40% degradation of MB in 21 min (Fig. 9c). The (0.05 : 1) and (0.075 : 1) samples reduce methylene blue within 13 and 1.5 min and degrade MB 68% and 89% respectively, and 0.1 : 1 sample accomplishes almost complete reduction (about 99%) of methylene blue to leuco-methylene blue (LMB) in 1 min at 25 °C. The successive decrease in absorption intensity of dye demonstrated a rapid reaction rate over a certain period.^52^ Besides, significant catalytic efficiency was observed for 0.1 : 1 sample (see Fig. 9g and h), suggesting that Zr-doped MoS_2_ is an excellent and efficient nanocatalyst for use in degradation of organic dyes.

**Fig. 10:**
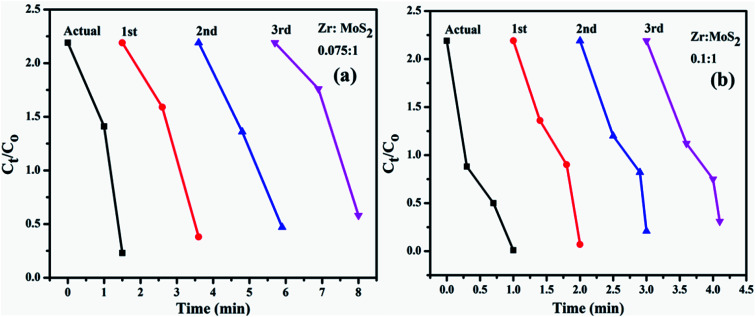
(a and b) Plots of *C*_*t*_/*C*_o_*versus* time for reusability of MoS_2_ nanosheets doped with 7.5 and 10 wt% Zr.

According to Beer Lambert's law, the ratio of the concentrated amount of MB at any time (*C*_*t*_) and initial concentrated amount (*C*_o_), namely *C*_*t*_/*C*_o_ can be evaluated using different concentrations of absorbance (*A*_*t*_/*A*_o_). Fig. 9g and 10 elucidate *C*_*t*_/*C*_o_ time course for prepared catalysts, which further indicates the catalytic reaction for catalysts used. Furthermore, reduction of MB was almost complete at the end of reaction period, indicating the important role played by Zr-doped MoS_2_. The dye degradation potential (measured in percentages) of all synthesized samples are shown in Fig. 9h, which was calculated using eqn (1):1
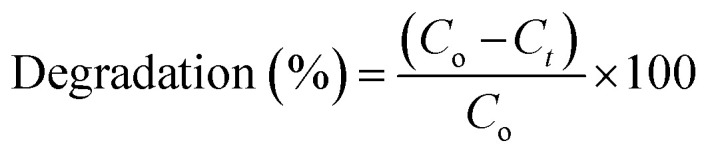


The pH value is an important factor in wastewater management. pH playes a vital role in the dye degradation reaction. In the present work, pH value was observed to be basic (7.9).

Stability also greatly influences the performance of catalysts during dye degradation reaction. To check the stability of catalysts, both activities were retained for 48 hours, after which duration the same results were observed as before. As degradation remained undisturbed, it indicated high stability of the catalysts. In the present work, reusability or recyclability of catalyst was assessed by recycling 7.5 and 10 wt% of Zr-doped MoS_2_. These particular catalyst samples were selected since they outperformed others in earlier experiments. The selected samples were examined for three cycles as can be seen in Fig. 10a and b. Furthermore, the load of catalyst was also verified before and after three recycling procedures. In Fig. 10a and b small weight loss of catalyst was detected *i.e.*, ranging from 2 mg (before) to 1.6 mg (after three cycles) as determined by deliberating ∼5% sensing deviation. From the above findings, Zr-doped MoS_2_ catalysts are considered to be highly stable during catalytic reactions showing remarkable catalytic potential for use in the treatment of industrial wastewater.

**Fig. 11:**
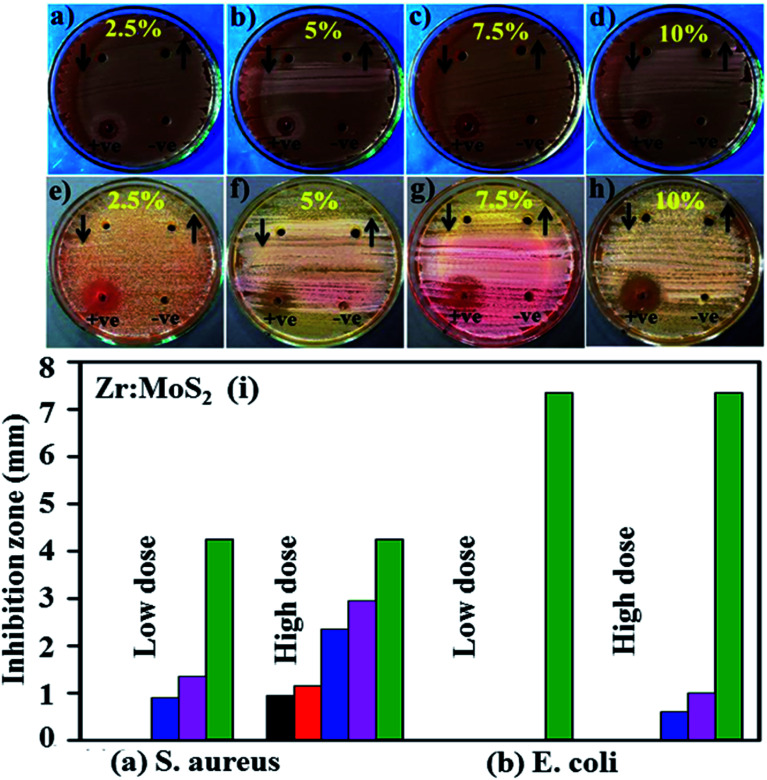
(a) *In vitro* antimicrobial efficacy of Zr-doped MoS_2_ (2.5%), (b) (5%), (c) (7.5%), (d) (10%) for *E. coli* and (e–h) for *Staph. aureus*, respectively and (i) comparative analysis.

The *in vitro* antibacterial efficacy of Zr-doped MoS_2_ nanosheets through agar well diffusion method against *E. coli* and *S. aureus* are shown in Fig. 11a–h and Table 1. The images depict enhanced antibacterial efficacy of Zr-doped MoS_2_ against *E. coli* in Fig. 11a–d compared with *S. aureus* in Fig. 11e–h. The results depict synergism between the concentrations of doped material and inhibition zones formed for G −ve. Statistically, significant inhibition zones were recorded against G −ve ranging from (0–1.35 mm) and (0.95–2.95 mm) at low and high concentrations of Zr-doped MoS_2_ and (0–1 mm) at high concentration against G +ve. A low concentration of doped material showed zero antibacterial efficacy for *S. aureus*. Ciprofloxacin as positive control showed 4.25 mm and 7.35 mm inhibition zone diameters against *E. coli* and *S. aureus*, respectively, compared with DIW (0 mm). Overall, Zr-doped MoS_2_ with 7.5 and 10 wt% dopant at low and high doses demonstrated significant antibacterial activity against G −ve (*E. coli*) compared with G +ve (*S. aureus*) as shown in Fig. 11a–h while, 2.5 and 5 wt% doping showed null activity against both bacteria at low concentrations as can be seen in comparative analysis exhibited in Fig. 11i. Oxidative stress of fabricated nanostructures depends upon the size and concentration of nanoparticles. It is know that the size of nanoparticles influences anti-bacterial efficacy.^53,54^ Small-sized particles generate reactive oxygen species (ROS) which enclose bacterial cell membrane through extrusion of cytoplasmic contents, resulting in bacterial death. Other possibilities for the reaction of nanomaterials with bacterial strains include strong interaction of cations with negatively charged parts of bacterial cells resulting in its collapse.^55,63,64^

**Table 1:** Antimicrobial activity of Zr-doped MoS_2_ nanosheets

Sample	Inhibition zone^a^ (mm)		Inhibition zone^b^ (mm)	
	0.5 mg/50 μl	1.0 mg/50 μl	0.5 mg/50 μl	1.0 mg/50 μl
2.50%	0	0.95	0	0
5%	0	1.15	0	0
7.50%	0.9	2.35	0	0.6
10%	1.35	2.95	0	1
Ciprofloxacin	4.25	4.25	7.35	7.35
DIW	0	0	0	0

a Inhibition zone (mm) of Zr-doped MoS_2_ nanosheets for *E. coli*.

b Inhibition zone measurements of Zr-doped MoS_2_ nanosheets for *Staph. aureus*.

## 4. Conclusion

The Zr-doped MoS_2_ nanosheets were successfully synthesized through hydrothermal route. Doping of nanosheets was performed with an aim to increase its antimicrobial and catalytic efficiency. The formation of hexagonal phase of MoS_2_ was confirmed through XRD analysis. Hexagonal MoS_2_ possessed an interlayer spacing of ∼0.27 nm, which matched well with the results obtained from HR-TEM. FTIR spectra indicated molecular bonding of Mo and S with functional groups, while characteristic transmittance of MoS_2_ was observed at ∼903 and 1124 cm^−1^. The surface morphology with an agglomeration of stacking layers was confirmed with FESEM and further evidence about interlayer spacing was attained through HR-TEM with ring feature of prepared specimens. Molecular fingerprint with vibrational modes of E_2g_ and A_1g_ confirmed the presence of in-plane vibrations of S atoms with respect to Mo atoms and S atomic vibrations in opposite direction to Mo atoms, respectively. It was suggested that the role of Zr in facilitating electron migration and recombination of charge was diminished and efficiency of electron mobility was increased in doped samples. UV-vis spectroscopy was performed and an absorption band in the range of 225 nm with redshift was observed. The present research confirmed that higher concentrations (0.1 : 1) of Zr-doped MoS_2_ nanosheets show excellent antibacterial efficacy and unique catalytic response such that it degrades 99% MB in just 1 min. Based on the present study, MoS_2_ nanosheets synthesized with specific Zr dopant concentrations have proved to be a novel material with outstanding antimicrobial and catalytic efficacy for use in the area of water purification and biomedical applications.

## Availability of data and materials

All data are fully available without restriction.

## Conflicts of interest

The authors declare that they have no competing interests.

## Supplementary Material
